# Wearable Sensors for Measurement of Viewing Behavior, Light Exposure, and Sleep

**DOI:** 10.3390/s21217096

**Published:** 2021-10-26

**Authors:** Khob R. Bhandari, Hanieh Mirhajianmoghadam, Lisa A. Ostrin

**Affiliations:** University of Houston College of Optometry, Houston, TX 77204, USA; krbhanda@central.uh.edu (K.R.B.); hmirhaji@central.uh.edu (H.M.)

**Keywords:** light exposure, near work, myopia, viewing behavior, wearable sensors

## Abstract

The purpose of this study was to compare two wearable sensors to each other and to a questionnaire in an adult population. For one week, participants aged 29.2 ± 5.5 years (*n* = 25) simultaneously wore a Clouclip, a spectacle-mounted device that records viewing distance and illuminance, and an Actiwatch, a wrist-worn device that measures illuminance and activity. Participants maintained a daily log of activities and completed an activity questionnaire. Objective measures of time outdoors, near (10–< 60 cm) and intermediate (60–100 cm) viewing, and sleep duration were assessed with respect to the daily log and questionnaire. Findings showed that time outdoors per day from the questionnaire (3.2 ± 0.3 h) was significantly greater than the Clouclip (0.9 ± 0.8 h) and Actiwatch (0.7 ± 0.1 h, *p* < 0.001 for both). Illuminance from the Actiwatch was systematically lower than the Clouclip. Daily near viewing duration was similar between the questionnaire (5.7 ± 0.6 h) and Clouclip (6.1 ± 0.4 h, *p* = 0.76), while duration of intermediate viewing was significantly different between methods (*p* < 0.001). In conclusion, self-reported time outdoors and viewing behaviors were different than objective measures. The Actiwatch and Clouclip are valuable tools for studying temporal patterns of behavioral factors such as near work, light exposure, and sleep.

## 1. Introduction

Outdoor illumination, near work, and sleep have been shown to be associated with eye growth and myopia (nearsightedness). Studies show that increased time outdoors in high intensity light is protective against myopia onset in children [1,2,3,4]. Rose et al. reported the highest prevalence of myopia among children who spent less time outdoors and a greater amount of time doing near work [5]. Another study reported greater progression of myopia in winter compared to summer in schoolchildren, which was suggested to be due to exposure to decreased time outdoors accompanied by increased academic pressure during the winter months [6]. Protective effects of time outdoors for myopia onset have also been reported in randomized controlled trials in children [4,7]. Given that light exposure has been linked to myopia, and light is the most potent cue for circadian rhythm, speculation exists whether circadian rhythms and sleep/wake patterns also play a role in myopia. Environmental light information is carried by intrinsically photosensitive retinal ganglion cells to the suprachiasmatic nucleus, the master clock of the body [8,9], where higher order pathways control diurnal release of various neurotransmitters and hormones, including melatonin [10,11,12]. Melatonin is integral in mediating sleep/wake patterns [13]. Recent studies report associations between various sleep parameters, melatonin, and myopia [14,15].

Numerous studies have also linked increased daily near work with myopia prevalence [16,17,18]. Studies in schoolchildren have reported that faster progressing myopia is associated with a shorter working distance [19,20]. However, results regarding the influence of near work on myopia pathogenesis are inconsistent, with some studies reporting no significant increased risk of myopia with increased near work [21,22,23]. The discrepancy across studies regarding the role of near work in myopia could be due to the subjective nature by which near work is assessed and quantified.

With accumulating evidence that behaviors contribute to myopia onset and progression, it is important to objectively and precisely quantify these factors [24,25,26,27,28]. Information regarding light exposure and near work are traditionally inferred from questionnaires, which are subject to errors in estimation and vulnerable to parental and recall biases [29,30]. Reports of daily and weekly activities from children and their parents have been shown to have a poor agreement [31], and subjective measures of time outdoors are often in poor agreement with objective measures [32,33,34]. Surrogate methods are often used to quantify near work, such as level of education [35], number of books read [36], and occupation [37], but these metrics fail to provide the full profile of viewing behavior. Viewing breaks and absolute viewing distance, which are difficult to quantify subjectively, may be important parameters to consider in myopia studies [38].

Though calibrated photometers and radiometers can provide more accurate measures of ambient illuminance, the practical utility of using them is often limited by cost, feasibility, and convenience. The unpredictability in human behaviors and frequent changes in light exposure from indoor to outdoor environments makes it more challenging to characterize exposure patterns in individuals with reasonable accuracy. Ambient illuminance ranges from less than 1 lux indoors to greater than 150,000 lux outdoors [33,39,40]. Hence, precise quantification of light exposure over a broad range of available light intensities requires robust objective tools with a large operating range. Ambulatory light sensors and actigraphy devices are more commonly being used to measure light exposure in both adults and children, with relative ease of wear and long battery life providing data over days or weeks to gather naturalistic behaviors [32,41,42,43,44]. Light sensors are often worn around the neck or on the wrist, which may not directly represent the amount of light entering the eye [45]. A recent study by Joyce et al. assessed the utility of two commercially available wrist-worn light sensors—the Philips Actiwatch 2 (Philips Respironics, Philips, Murrysville, PA, USA) and the Activinsights GENEActiv (Activinsights, Cambridgeshire, UK)—and reported good performance in monitoring the temporal pattern of light intensity, but lower accuracy in illuminance-sensing under moderately intense natural and artificial lighting, usually underestimating true light levels [46]. The Clouclip is a small, battery-powered light sensor and range-finding device that was developed for use in myopia research [47]. The Clouclip is mounted to the temple of a spectacle frame [47,48,49,50]. Because the Clouclip is positioned at eye level, the measured illumination may be more representative of light reaching the eye compared to sensors worn on the arm or around the neck, such as the Actiwatch or HOBO light monitor [32,34,42]. The Clouclip has the additional advantage of not only measuring illuminance, but also measuring viewing distance, so that both light exposure and near work can be quantified with a single instrument.

The goal of the current study was to use subjective and objective methods to quantify environmental and behavioral risk factors related to myopia. Specifically, the study aimed to compare objective measures of light exposure and time outdoors using two wearable sensors, the Clouclip and Actiwatch, to each other and to a questionnaire. An additional aim was to compare near viewing and sleep duration between objective data from the sensors and subjective data from the questionnaire. Quantification of near work and light exposure is important as they are commonly studied risk factors for myopia. This study is novel as it uses a new wearable sensor, the Clouclip, to objectively quantify light exposure and near viewing behaviors in a habitual work environment over a week-long period. Results from the study show a good utility of objective sensor devices in recording personal light exposure, with added quantification of near viewing behaviors using Clouclip. Findings support the use of these devices in further research investigating risk factors for myopia.

## 2. Materials and Methods

### 2.1. Participants

Young adults were recruited for this study. All participants had 20/25 or better visual acuity with habitual refractive correction and no history of ocular disease. Ethical approval was obtained from the Committee for Protection of Human Subjects at the University of Houston, and procedures adhered to the tenets of the Declaration of Helsinki. All participants provided written consent prior to the participation in the study. Data were collected between February and April 2021.

Baseline measures included distance visual acuity, ocular biometry, and non-cycloplegic autorefraction. For biometry (LenStar LS900, Haag-Streit, Switzerland) and non-cycloplegic refractive status (WAM-5000, Grand Seiko, Japan), five measurements were taken in each eye and averaged. Participants were classified based on their non-cycloplegic spherical equivalent refractive error as being myopic (mean spherical equivalent refractive error of right and left eyes ≤ −0.50 DS, with at least one eye measuring 0.75 DS or more myopia) or non-myopic (mean spherical equivalent refractive error from right and left eyes of +1.25 to −0.50 DS, with no eye exhibiting 0.75 D or greater myopia). Participants with hyperopia (spherical equivalent refractive error ≥ +1.50 D) were excluded to avoid potential confounding effects of this refractive error on viewing behavior.

### 2.2. Instrumentation

**Figure 1:**
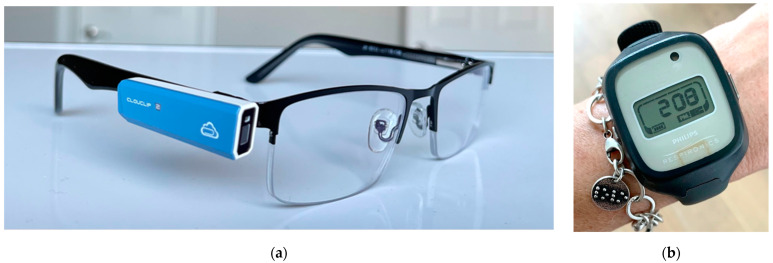
(**a**) Clouclip device fitted to the right temple of a spectacle frame; (**b**) Actiwatch Spectrum Plus.

This study utilized 15 Clouclips (Glasson Technology Co Ltd., HangZhou, China) for objective measurement of ambient illuminance and viewing distance, and 16 Actiwatches (Actiwatch Spectrum Plus; Philips Respironics, Murrysville, PA, USA) for objective measurement of ambient illuminance and sleep (Figure 1). Characteristics of each instrument are presented in Table 1. Clouclips used in this study were tested for distance and illuminance measurement, and Actiwatches were tested for illuminance measurement, as previously described [33,51].

The Clouclip is a small, lightweight Bluetooth device that is mounted to the right temple of a spectacle frame. The Clouclip measures distance every 5 s using infrared tracking beam and ambient illuminance every 2 min using a light sensor [52]. The reported diameter of the tracking beam (i.e., field of view of the distance sensor) is 25 degrees, with a measurement range of 5–120 cm. The reported range for illuminance is 1–65,336 lux. The device has angular acceleration sensors (X, Y, and Z axes); if no movement is detected for 40 s the device goes into sleep mode for a minimum of two minutes and until movement is detected again. Data are stored in the device, then uploaded to a cloud using Bluetooth connection and a smartphone application. The Clouclip must be charged nightly. Once fully charged, the Clouclip can record data continuously throughout a day. Over one week’s data can be stored in the Clouclip. The Clouclip has been previously validated for distance and illuminance [49,51].

The Actiwatch Spectrum Plus is a wrist-worn actigraph device that measures ambient illuminance and activity at 32 Hz. The Actiwatch contains color-sensitive silicone-photodiodes to measure visible light illuminance (wavelength 380–750 nm), with a measuring range of 0.1–35,000 lux for white light. Wearers must ensure their sleeve is not obstructing the sensor on the watch for accurate measures of illuminance. The Actiwatch Spectrum Plus has a MEMS-type accelerometer to measure physical activity, expressed in counts per minute (cpm). The Actiwatch is light weight (31 g) and waterproof for up to 30 min, allowing for continuous wear. Actiwatches were fully charged and configured to average over one-minute epochs, which allows data storage up to 50 days. The Actiwatch contains an “off-wrist” sensor to monitor wear compliance. The Actiwatch has been previously validated for physical activity [53] and sleep parameters [54].

### 2.3. Procedures

Following baseline measures, participants were asked to wear the Clouclip and Actiwatch simultaneously for 7 days, including 5 weekdays and 2 weekend days. The Clouclip was worn during waking hours, except for showering or swimming, and the Actiwatch was worn continuously. Participants were instructed to make a note on a daily log if the devices had to be removed. Each participant completed the UH Near Survey Supplementary Materials to answer questions related to their near work activities, outdoor time, physical activity, and sleep during the experimental week. The questionnaire asks participants to estimate their time spent in the various activities separately for weekdays and weekends. Participants also kept a daily log of near activities for two days, one weekday and one weekend day, to directly compare logged activities with objective measures.

The Clouclip was fitted to the right temple of the participant’s habitual spectacles or of provided glasses with plano (zero powered) lenses. The Actiwatch was worn on the non-dominant wrist. After a week of wearing both devices, participants returned the Clouclip, Actiwatch, completed questionnaire, and activity log to the lab. Clouclip-recorded data were uploaded to a cloud using Bluetooth connection and the custom smartphone application. Date- and time-tagged data from the cloud were downloaded and analyzed using a custom MATLAB program. Actiwatch-recorded data were uploaded to the Actiware software (Actiware Version 6.0.9; Philips Respironics, Murrysville, PA, USA).

### 2.4. Data Analysis

Questionnaire-derived time outdoors was determined from the number of hours the participants estimated spending outdoors in leisure activity, sports, and driving. Sleep duration was derived from participants’ estimates of the amount of time they sleep per night. Near viewing was determined from the number of hours the participants estimated spending on various near activities, including reading printed material, drawing, painting, writing, and using hand-held devices. Intermediate viewing was determined from the number of hours spent using computers and playing board games or cards. Subjective diopter hours were calculated from questionnaire data using Equation (1). Activities performed at the closest distance were weighted times 3, as previous studies show that these activities are performed at distances from approximately 25–45 cm, or mean dioptric demand of, on average, 3 diopters (D) [44,51]. Activities performed at an intermediate distance were weighted times 1.5, as these activities are generally performed at distances of 60–100 cm, or dioptric demand of approximately 1.5 D [44]. Weekday and weekend diopter hours were calculated separately.

Subjective diopter hours = [3 × (hours reading print + hours drawing, painting, writing + hours using handheld devices)] + [1.5 × (hours using computers, playing board games or cards)](1)

Clouclip data were analyzed using a custom MATLAB program. For each participant, the total wear time for each day was determined; if the Clouclip recorded valid data for ≥80% of the total wake-time, that day was considered “valid.” For a participant to be included in the analysis, data for at least three valid weekdays and one valid weekend day were required. Clouclip-measured illuminance was analyzed for time spent outdoors (minutes exposed to ≥1000 lux) per day and mean daily light exposure. Clouclip-measured viewing distances were analyzed for minutes of near (10 cm to <60 cm) and intermediate (60 cm to <100 cm) viewing per day. Objective diopter hours were calculated from Clouclip-recorded data for weekdays and for weekend days using Equation (2):

Objective diopter hours = [3 × (hours viewing from 0.1 m to <0.6 m)] + [1.5 × (hours viewing 0.6 m to <1 m)](2)

Data from the Actiwatch were uploaded to the Actiware software. Light exposure data from the Actiwatch were included only if the day was considered valid for the Clouclip data so that direct comparisons could be carried out. Actiwatch data were analyzed for (1) time outdoors (minutes exposed to ≥ 1000 lux) per day, (2) mean daily light exposure (lux), and (3) sleep duration (hours per night).

Because behaviors have been shown to vary between weekdays and weekend days, each near work and light exposure metric was averaged separately for weekdays (Monday–Friday) and for weekend days (Saturday–Sunday) [39,55]. Sleep duration was calculated for weeknights (Sunday–Thursday nights) and weekend nights (Friday–Saturday nights). From this, a single mean daily value was calculated using Equation (3):

Mean daily metric = [(average weekday metric × 5) + (average weekend metric × 2)]/7(3)

Statistical analysis was performed in SPSS 22.0 (IBM Corp., Armonk, NY, USA). Results are presented as mean ± standard error unless otherwise noted. Repeated measure ANOVAs were performed with two within-subject factors, method of data collection (Clouclip, Actiwatch, and questionnaire) and day of the week (weekday and weekend day). Post-hoc Bonferroni corrected pairwise comparisons were carried out when significant main effects or interactions were observed. Linear regression and Bland–Altman analyses were performed to compare the differences in mean daily light exposure recorded with the Clouclip and Actiwatch.

## 3. Results

Twenty-five participants (11 males, 14 females) with mean (± standard deviation) age 29.2 ± 5.5 years (range: 22–45 years) completed the study. Eight participants were classified as emmetropic and 17 as myopic. Spherical equivalent refractive error and axial lengths were similar between right and left eyes (*p* = 0.32 and *p* = 0.10, respectively), so only right eyes are included further. Mean (± standard deviation) spherical equivalent refractive error was −2.12 ± 0.24 D (range +0.25 to −6.50 D) and axial length was 24.40 ± 1.33 mm (range 22.48 to 27.64 mm).

**Figure 2:**
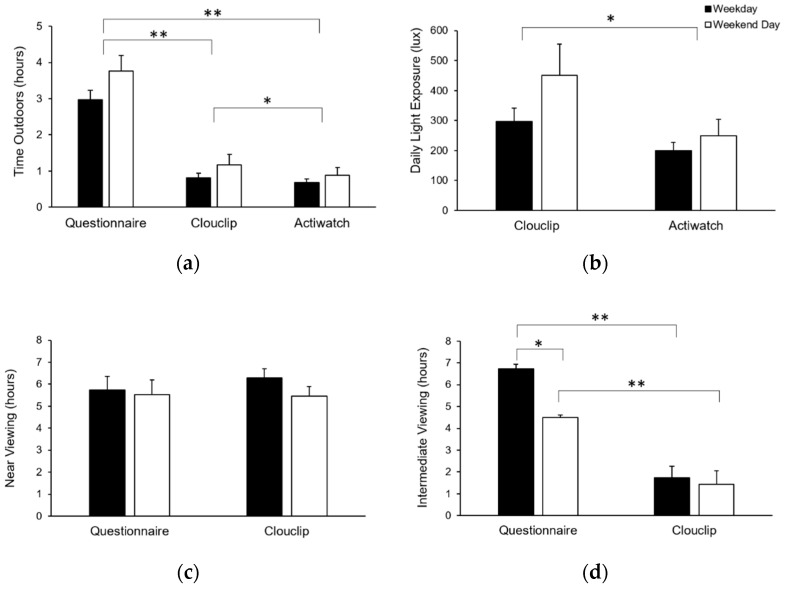

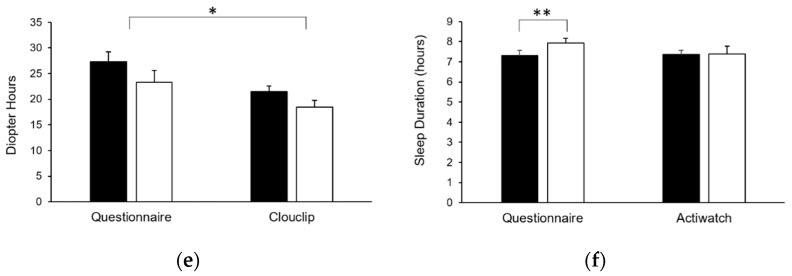
(**a**) Time outdoors (hours), (**b**) daily light exposure (lux), (**c**) near viewing (hours), (**d**) intermediate viewing (hours), (**e**) diopter hours, and (**f**) sleep duration on weekdays (filled bars) and weekend days (open bars) derived from the questionnaire, Clouclip, and Actiwatch; asterisks (*) represent statistically significant differences determined by repeated measures ANOVAs, * *p* < 0.05, ** *p* < 0.001.

Mean daily values for all metrics calculated from the questionnaire, Clouclip, and Actiwatch are presented in Table 2, and values separated by weekday and weekend days are shown in Figure 2. All participants had four or more valid days and were included in all analyses. The mean number (± standard deviation) of valid Clouclip wear days included in the analysis was 6.8 ± 0.6 (range 4–7). Mean daily Clouclip wear time was 14.9 ± 1.7 h, with no difference in wear time between emmetropes and myopes (14.4 ± 0.7 h and 14.6 ± 2.1 h, respectively, *p* = 0.88).

### 3.1. Light Exposure

Time outdoors was determined subjectively from the questionnaire and obtained objectively from the Clouclip and Actiwatch. According to the questionnaire, participants spent 3.0 ± 0.3 h and 3.8 ± 0.4 h outdoors per day on weekdays and on weekend days, respectively, with a mean daily total of 3.2 ± 0.3 h. Time outdoors as measured with the Clouclip was 0.8 ± 0.1 h on weekdays and 1.2 ± 0.3 h on weekends, with a mean daily total of 0.9 ± 0.2 h. Time outdoors as measured with the Actiwatch was 0.7 ± 0.1 h on weekdays and 0.9 ± 0.2 h on weekend, with a mean daily total of 0.7 ± 0.1 h. Repeated measures ANOVA showed main effects of both method of collection (*p* < 0.001) and day of the week (*p* = 0.03). Post-hoc comparisons showed that estimates of time outdoors from the questionnaire were significantly higher than objective measures from the Clouclip (*p* < 0.001) and Actiwatch (*p* < 0.001). Time outdoors measured by the Clouclip was significantly greater than the Actiwatch (*p* = 0.02). Across all methods, time outdoors was greater on weekends compared to weekdays.

**Figure 3:**
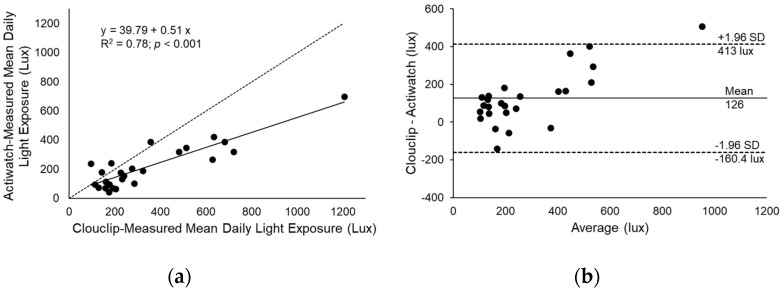
(**a**) Linear regression (solid line) between mean daily light exposure measured with Clouclip and Actiwatch; dashed line represents 1:1 line, and (**b**) Bland–Altman analysis of mean daily light exposure measured with the Clouclip and Actiwatch; solid line represents the mean difference and dashed lines represent the limits of agreement.

Clouclip-measured daily light exposure for weekdays was 299 ± 45 lux and for weekend days was 451 ± 104 lux, with a mean daily light exposure of 342 ± 52 lux. Actiwatch-measured daily white light exposure for weekdays was 200 ± 27 lux and for weekend days was 250 ± 54 lux, with a mean daily white light exposure of 215 ± 31 lux. Daily white light exposure was correlated between the two devices (R^2^ = 0.78, *p* < 0.001). Bland–Altman analysis showed that daily white light exposure from the Actiwatch was systematically lower than the Clouclip (mean difference 126 lux, limits of agreement −160 to 413 lux, Figure 3).

### 3.2. Near Viewing

Near viewing duration from the questionnaire for weekdays and weekend days was 5.7 ± 0.6 h and 5.5 ± 0.7 h, respectively, with a daily mean duration of 5.7 ± 0.6 h. Clouclip-derived daily duration of near viewing for weekdays and weekend days was 6.3 ± 0.4 and 5.4 ± 0.4 h, respectively, with a daily mean duration of 6.1 ± 0.4 h. There was a significant main effect of day of the week (*p* = 0.03), but not method of collection (*p* = 0.76); near viewing duration was greater on weekdays compared to weekend days.

Intermediate viewing duration from the questionnaire for weekdays and weekend days was 6.7 ± 0.5 and 4.5 ± 0.6 h, respectively, with a daily mean duration of 6.1 ± 0.5 h. Clouclip-derived daily duration of intermediate viewing for weekdays and weekend days was 1.7 ± 0.2 and 1.4 ± 0.1 h, respectively, with a daily mean duration of 1.6 ± 0.2 h. Intermediate viewing duration showed a significant main effect of day of the week (*p* < 0.001) and method of collection (*p* < 0.001). There was a significant interaction between day of the week and method of collection (*p* < 0.001). Questionnaire-derived intermediate viewing duration was greater than the Clouclip for both weekdays (*p* < 0.001) and weekends (*p* < 0.001). Additionally, from the questionnaire, intermediate viewing on weekdays was greater than weekend days (*p* < 0.001).

Subjective diopter hours from the questionnaire for weekdays and weekend days was 27.3 ± 1.9 dh and 23.8 ± 2.4 dh, respectively, with a mean daily total of 26.1 ± 2.0 dh. Clouclip-derived objective diopter hours for weekdays and weekend days were 21.5 ± 1.1 dh and 18.5 ± 1.33 dh, respectively, with a mean daily total of 20.6 ± 1.1 dh. Diopters showed a significant main effect of day of the week (*p* < 0.001) and method of collection (*p* = 0.02). Diopter hours were greater for weekdays than weekend days (*p* < 0.001), and subjective diopter hours were greater than objective diopter hours (*p* = 0.02).

### 3.3. Sleep Duration

Sleep duration from the questionnaire for weeknights and weekend nights was 7.3 ± 0.3 h and 7.9 ± 0.3 h, respectively, with a mean daily duration of 7.5 ± 0.2 h. Actiwatch-derived sleep duration for weeknights and weekend nights was 7.4 ± 0.2 h and 7.4 ± 0.4 h, respectively, with a mean daily duration of 7.4 ± 0.3 h. Sleep duration showed a main effect of day of week (*p* = 0.02), but not method of collection (*p* = 0.39). A significant interaction followed by post-hoc comparison showed that the questionnaire-derived sleep duration was greater on weekend nights than weeknights (*p* = 0.001).

## 4. Discussion

Objective wearable sensors allow for characterization of temporal patterns and absolute values of behaviors, such as timing, intensity, and duration, in the wearer’s habitual environment. This study compared measures of time outdoors and light exposure between two objective wearable sensors, the Clouclip and Actiwatch, over a one-week period. Near and intermediate viewing were quantified with the Clouclip, and sleep duration was quantified with the Actiwatch. Objective measures were then compared to subjective measures from a questionnaire, and behaviors were compared across weekdays and weekend days. Results show that participants tended to overestimate time spent outdoors. Although self-reported time spent in near viewing closely matched objective measures, time spent in intermediate viewing were overestimated compared to objective measures. Self-reported sleep duration was not significantly different than objective measures from the Actiwatch. Participants spent more time outdoors and less time in near viewing on weekend days compared to weekdays. Illuminance measurement was correlated between the Clouclip and Actiwatch; however, Actiwatch measures were systematically lower than the Clouclip by about 126 lux.

The purpose for studying the Clouclip and Actiwatch was the broad operational ranges of these instruments, which are relevant to myopia research and the relative ease of wear over extended period. Both instruments are objective and continuously measure the parameters of interest with little to no effort from the participants. In addition to ambient light sensing, the Actiwatch provides data on physical activity and sleep duration, and the Clouclip records near work distances in a reasonable sampling frequency to be able to objectively quantify near viewing behaviors. Moreover, there are few commercially available range finders that are portable and suitable for ambulatory measurement.

Self-reported overestimates in time outdoors have been previously reported in both adults and children [33,40]. In the questionnaire utilized here, the UH NEAR Survey, participants were asked to estimate the amount of time they spent in outdoor light, whether in sports, leisure activity, or driving or riding in a car, to encompass all high intensity light exposure. Time outdoors as determined from objective measures is commonly calculated as minutes per day exposed to greater than 1000 lux [33,39]. Participants overestimated their time outdoors by approximately two hours per day, highlighting the need for objective measures for accurate measures of time spent outdoors, as it is well known that questionnaires are subject to errors in estimation and vulnerable to recall biases [29,30]. Furthermore, the questionnaire used here had limited resolution (half-hourly), which limited the precision that could be obtained from the questionnaire.

Both the Clouclip and Actiwatch have been validated for measuring illuminance [33,47,51] and have been used previously in myopia studies [47,48,49,50]. Here, findings show that illuminance measures were highly correlated between the two devices. However, the Actiwatch-measured illuminance was systematically lower than the Clouclip. The Clouclip is worn on the right temple of a spectacle frame with the light sensor directed forward, along the axis of the head. On the other hand, the Actiwatch is located on the wrist and is subject to greater variation in the orientation of the light sensor. A previous study recorded 24 h illuminance at eye level and the wrist and reported a correlation of R = 0.76 between the two locations [56]. Studies show that the Actiwatch is sensitive to orientation [41,57], which likely contributes to the increased variability. The Actiwatch is also subject to obstruction by the wearer’s sleeve. Another factor that may have contributed to lower illuminance measures with the Actiwatch is the upper cut-off of the sensor. Manufacturer-reported upper cut-off is 35,000 lux for the Actiwatch and 65,336 lux for the Clouclip. Outdoor illuminance has been shown to reach over 150,000 lux in direct sunlight [40]. Therefore, while both sensors are capable of quantifying time outdoors (≥1000 lux), absolute illuminance measures may be underestimated because of the ceiling effect. Given that the Clouclip is placed close to the eyes, oriented in a similar direction as the line of sight, and has a higher upper range, the Clouclip may be a more appropriate sensor to measure eye-level illuminance when photopic measures are of interest. On the other hand, the lower cut off also varies between devices; the Actiwatch has a lower cut off of 0.1 lux, whereas the Clouclip has a lower cut off of 1 lux. Therefore, if the metric of interest is in the scotopic or lower mesopic range, the Actiwatch may be more appropriate. With increasing interest in the role of low indoor lighting in childhood myopia, it is important to have reliable objective devices with an operating range that can capture the light levels of interest [58].

Subjective measures of near work in this study were obtained from the questionnaire. Recently, several devices have been introduced for objective measurement of near work, including the Clouclip [47,48,49,50], Vivior [59,60], and RangeLife [44]. Near viewing includes a range of activities with varying distances, such as reading, writing, computer use, and handheld electronics. Subjective estimation is challenging, as it is difficult to estimate duration of near work episodes and absolute viewing distance. Interestingly, in this study, subjective estimates of daily near viewing were similar to objective measures from the Clouclip (10 to < 60 cm). However, subjective estimates and objective measures of intermediate viewing (60 to < 100 cm) were significantly different. One potential source of this discrepancy is how activities were classified. For example, we considered computer use to be an intermediate viewing activity in the questionnaire, whereas some participants may view their computer monitor as close as 40 cm, which would be classified as near viewing from the Clouclip. Validation studies in our lab show that the Clouclip measures distances up to 120 cm; however, it becomes less reliable with distances > 100 cm as the tracking beam diameter is greater, and the intensity of the reflected beam is lower [51]. The Clouclip records the viewing distance for the nearest target that reflects the tracking beam, which might limit the quantification of viewing distances in a crowded visual environment that has targets within close proximity to each other. Another source of the discrepancy between subjective and objective measures of near and intermediate viewing is viewing breaks; objective continuous measures are sensitive to viewing breaks. If a participant is performing a near task and occasionally looks away from the material, the viewing break will not be included in the total duration of near viewing. For this reason, objective measures with high resolution, such as that obtained with the Clouclip every 5 s, will more accurately represent the temporal patterns of viewing behavior than subjective estimates.

While wearable sensors are powerful tools for objective and continuous measurement of human behaviors, they do present limitations, which may ultimately lead to errors in the data. The Clouclip is mounted on the temple of glasses, and therefore requires spectacles to be worn. This necessitates emmetropic participants to wear glasses for the duration of the measurement period, which has the potential to bias behaviors. In recording viewing distances, the Clouclip is aligned with the axis of the head and not with the line of sight of the eyes, which may introduce errors in measurement as the eyes rotate. Additionally, the Clouclip is not waterproof and cannot be worn during the night. Therefore, there are gaps in data collection. Limitations of the Actiwatch include the relatively distal location of the sensor from the eyes and variations in orientation as the arm moves in space. There is the potential for the sensor to be obstructed by clothing, which will result in a light measurement that is not truly experienced by the eyes. Measured light exposure may also be limited by the measurement range of each device. Additionally, when considering the response properties of the human eye, describing light exposure as log values may be a more relevant metric for quantification of human light exposure. Errors may also occur in estimation of sleep duration, as often, individuals have difficulty distinguishing the time that is spent asleep versus awake while in bed. Therefore, utilizing an objective sensor such as the Actiwatch can more accurately account for sleep latency and time awake in bed.

With current technology, no one sensor can capture all behaviors relevant to myopia. Here, near viewing was recorded only by the Clouclip, and activity/sleep were recorded only by the Actiwatch. Even with both devices, there are still many unknown factors, such as what the near viewing targets may be. For example, the Clouclip cannot distinguish between a printed book and handheld electronic device. Such distinction is of importance, as studies suggest that electronic device use may contribute to myopia [61,62,63]. In order to capture the whole visual environment, questionnaires must still be utilized along with sensors.

Here, both myopic and non-myopic participants were included so that a broad range of potential behaviors could be evaluated, although the population was too small to analyze behaviors by refractive error group. Previous studies have reported that behaviors are similar between myopic and non-myopic adults [32,33], so even with a larger population of adults, we may not observe differences in behaviors by refractive error group. One week may not be long enough to fully capture behaviors; therefore, future studies should consider employing wearable sensors for a longer time period or across various times of the year. However, the goal of this study was to compare measurements obtained with different instruments (the Clouclip, Actiwatch, and questionnaire), not to evaluate behaviors by refractive error group. Our ongoing and future studies are aimed at using these objective sensors to quantify behaviors by refractive error in children, the population of interest when studying myopia onset and progression.

## 5. Conclusions

In conclusion, the Clouclip and Actiwatch showed good utility and correlation in recording illuminance and time outdoors objectively in adult participants over a one-week period. The Clouclip was able to objectively quantify near viewing behaviors with high resolution, showing that young adults spend approximately 6 h in near viewing per day. Subjective estimates of time spent outdoors were significantly higher than objective measures. Subjective and objective estimates of daily near plus intermediate viewing duration were also in poor agreement, whereas subjective and objective estimates of sleep duration were similar. Given the ease of wear, high temporal measurement resolution, and increased accuracy compared to using only subjective methods, the Clouclip and Actiwatch are useful tools in myopia research to study temporal patterns of behavioral factors, such as light exposure, near work, and sleep. Findings from questionnaires and objective sensors complement each other to obtain a more comprehensive picture of an individual’s visual environment. Researchers now have instruments to conduct clinical investigations to objectively assess risk factors for myopia in children. Ultimately, findings will contribute to evidence-based recommendations for behavioral modifications regarding time outdoors and near work to decrease the risk for myopia.

## Figures and Tables

**Table 1:** Characteristics of the two sensors utilized in this study, the Clouclip and Actiwatch.

Parameter	Clouclip	Actiwatch
Model	Glasses clip M2	Spectrum Plus
Size	4.5 × 1.3 × 0.8 cm	4.8 × 3.7 × 1.5 cm (not including wristband)
Weight	5 g	31 g (including wristband)
Location	Spectacle mounted	Wrist-worn
Battery life	One day	Over 30 days
Communication	Bluetooth	MiniUSB
Parameters measured	Viewing distance (range 5–120 cm), illuminance (range 1–65,336 lux)	Physical activity, sleep, illuminance (range 0.1–35,000 lux)
Sampling frequency	0.2 Hz for viewing distance, 0.0083 Hz for illuminance	32 Hz, set to average over 1 min epochs
Memory capacity	Approximately 8 days	Over 30 days

**Table 2:** Mean daily time outdoors, light exposure, diopter hours, near and intermediate viewing, and sleep duration are shown for the questionnaire, Clouclip, and Actiwatch.

Parameter	Questionnaire	Clouclip	Actiwatch
Time outdoors (h)	3.2 ± 0.3	0.9 ± 0.8	0.7 ± 0.1
Daily light exposure (lux)	N/A	347 ± 10	215 ± 31
Time in near viewing (h)	5.7 ± 0.6	6.1 ± 0.4	N/A
Time in intermediate viewing (h)	6.1 ± 0.5	1.6 ± 0.2	N/A
Diopter hour (dh)	26.1 ± 2.0	20.6 ± 1.1	N/A
Sleep (h)	7.5 ± 0.2	N/A	7.4 ± 0.3

## Data Availability

Data will be made available upon request.

## Supplementary Materials

The following are available online at https://www.mdpi.com/article/10.3390/s21217096/s1, File S1: Activity survey.
