# Supplementary material for: Wearable Sensors for Measurement of Viewing Behavior, Light Exposure, and Sleep
Source: Sensors (Basel). 2021 Oct 26;21(21):7096. doi: 10.3390/s21217096 (PMC8587946; doi:10.3390/s21217096)
Supplement: Supplementary file 1 [file sensors-21-07096-s001.zip › sensors-1376302-supplementary.pdf]

Supplementary Material File S1. Activity Survey

Please estimate and mark the number of hours PER DAY you spend involved in these various activities for weekday (Monday-Friday) and a weekend day (Saturday-Sunday) for the CURRENT ONE WEEK PERIOD (sensor wear period)

For example, if you spend 3 hours on a weekday and 4.5 hours on a weekend day, please mark it like this:

Weekday: 0 1 2 3 4 5 6 7 8 9 10 11 12 | Weekend: 0 1 2 3 4 5 6 7 8 9 10 11 12

1. How many hours per day do you spend in <u>outdoor physical activity</u>, such as sports, hiking, walking, biking, running, etc.?

Weekday: 0 1 2 3 4 5 6 7 8 9 10 11 12 | Weekend: 0 1 2 3 4 5 6 7 8 9 10 11 12

2. How many hours per day do you spend in <u>outdoor leisure activity</u>, such as eating outdoors, sitting on a patio, etc?

Weekday: 0 1 2 3 4 5 6 7 8 9 10 11 12 | Weekend: 0 1 2 3 4 5 6 7 8 9 10 11 12

3. How many hours per day do you spend <u>driving or riding in vehicle</u>, such as a car, bus, train, etc?

Weekday: 0 1 2 3 4 5 6 7 8 9 10 11 12 | Weekend: 0 1 2 3 4 5 6 7 8 9 10 11 12

4. How many hours per day do you spend in <u>indoor physical activities</u> and sports, such as exercise, martial arts, indoor basketball, etc.?

Weekday: 0 1 2 3 4 5 6 7 8 9 10 11 12 | Weekend: 0 1 2 3 4 5 6 7 8 9 10 11 12

5. How many hours per day do you spend <u>participating in fine arts</u> (theatre, choir, orchestra, band, playing an instrument, etc.)

Weekday: 0 1 2 3 4 5 6 7 8 9 10 11 12 | Weekend: 0 1 2 3 4 5 6 7 8 9 10 11 12

6. How many hours per day do you spend viewing a television screen, whether you are watching shows or playing video games?

Weekday	Weekend
0 1 2 3 4 5 6 7 8 9 10 11 12	0 1 2 3 4 5 6 7 8 9 10 11 12

7. How many hours per day do you spend viewing a computer screen, whether you are working, browsing, watching shows, playing games, etc.?

Weekday	Weekend
0 1 2 3 4 5 6 7 8 9 10 11 12	0 1 2 3 4 5 6 7 8 9 10 11 12

8. How many hours per day do you spend viewing a handheld electronic device, such as a phone, tablet, or kindle, whether you are working, browsing, watching shows, playing games, etc.?

Weekday	Weekend
0 1 2 3 4 5 6 7 8 9 10 11 12	0 1 2 3 4 5 6 7 8 9 10 11 12

9. How many hours per day do you spend reading printed material, such as books, magazines, newspaper, work material, etc.?

Weekday	Weekend
0 1 2 3 4 5 6 7 8 9 10 11 12	0 1 2 3 4 5 6 7 8 9 10 11 12

10. How many hours per day do you spend writing, drawing, painting, or crafting?

Weekday	Weekend
0 1 2 3 4 5 6 7 8 9 10 11 12	0 1 2 3 4 5 6 7 8 9 10 11 12

11. How many hours per day do you spend playing cards or board games (not electronic)?

Weekday	Weekend
0 1 2 3 4 5 6 7 8 9 10 11 12	0 1 2 3 4 5 6 7 8 9 10 11 12

12. How many hours of sleep do you get each night?

Weekday	Weekend
0 1 2 3 4 5 6 7 8 9 10 11 12	0 1 2 3 4 5 6 7 8 9 10 11 12
